# BST2 negatively regulates porcine reproductive and respiratory syndrome virus replication by restricting the expression of viral proteins

**DOI:** 10.1016/j.virusres.2023.199181

**Published:** 2023-07-25

**Authors:** Yujiao Zhang, Ning Kong, Jinfeng Ti, Dongshen Cao, Zhaofeng Sui, Aimin Ge, Liuting Pan, Kuan Zhao, Yanjun Zhou, Guangzhi Tong, Liwei Li, Fei Gao

**Affiliations:** a Shandong Vocational Animal Science and Veterinary College, Weifang 261061, PR China; b Shanghai Veterinary Research Institute, Chinese Academy of Agricultural Sciences, Shanghai 200241, PR China; c College of Veterinary Medicine, Hebei Agricultural University, Baoding 071001, PR China; d Jiangsu Co-Innovation Center for the Prevention and Control of Important Animal Infectious Disease and Zoonose, Yangzhou University, Yangzhou 225009, PR China

**Keywords:** Prrsv, Bst2, E protein, Nsp12, Antiviral activity

## Abstract

Porcine reproductive and respiratory syndrome virus (PRRSV) has seriously affected the viability of swine industries worldwide, and effective measures to control PRRSV are urgently required. Understanding the mechanisms of action of antiviral proteins is crucial for developing antiviral strategies. Interferon-induced bone marrow stromal cell antigen 2 (BST2) can inhibit the replication of various viruses via different pathways. However, little is known about the effects of BST2 on PRRSV. Therefore, this study aimed to evaluate whether the interferon-induced BST2 can inhibit PRRSV replication. We used western blotting and RT-qPCR techniques to analyze the effect of BST2 overexpression and knockdown on PRRSV replication. Overexpression of BST2 inhibited the replication of PRRSV, whereas knockdown of BST2 by small interfering RNA promoted PRRSV replication. Additionally, the expression of BST2 was upregulated during the early phase of PRRSV infection in porcine alveolar macrophages. Analysis of PRRSV proteins showed that BST2 restricted the expression of several non-structural viral proteins. BST2 downregulated the expression of Nsp12 through a proteasome-dependent pathway and downregulated the expression and transcription of E protein. These findings demonstrate the potential of BST2 as a critical regulator of PRRSV replication.

## Highlights

• Bone marrow stromal cell antigen 2 (BST2) can inhibit viral replication.
• BST2 exhibits significant anti-PRRSV activity.
• BST2 expression is up-regulated during the early phase of infection.
• BST2 restricts the expression of viral proteins.

## 1 Introduction

Porcine reproductive and respiratory syndrome (PRRS) has been threatening the swine industry for over three decades. The etiological agent of PRRS is PRRS virus (PRRSV), an enveloped, non-segmented, single-stranded, positive-sense RNA virus classified in the Arteriviridae family within the Nidovirales order (Dokland, 2010). PRRSV strains are classified into two distinct genotypes, type-1 and type-2, which share 55–70% nucleotide identity (Lunney et al., 2016). The PRRSV genome is approximate 15 kb in length, encodes at least 11 open reading frames (ORFs), and contains more than 16 non-structural proteins and 8 structural proteins (Fang and Snijder, 2010). Deletions, insertions, and recombinations frequently occur in the PRRSV genome and lead to genetic diversity. Therefore, PRRS has become difficult to combat, and novel and effective control strategies are urgently required.

Commercially available vaccines have limited cross-protective efficacy against heterologous infections. Several studies have indicated that some cell-host factors can regulate the replication of viruses, such as CD163, sialoadhesin, heparin sulfate, vimentin, CD151, and CD209, as well as other cellular factors, such as MYH9, CH25H, annexin A2, and ZAP (An et al., 2020; Du et al., 2017; Ke et al., 2019; Zhao et al., 2020). Similarly, studies from our laboratory have shown that host factors, including PCSK9, MOV10, galectin-1, and PSMB1, restrict PRRSV replication via different pathways (Li et al., 2023, 2019; Zhang et al., 2020; Zhao et al., 2018).

Bone marrow stromal cell antigen 2 (BST2) contains an N-terminal cytoplasmic tail domain, a transmembrane (TM) domain, a coiled-coil (CC) ectodomain, and a C-terminal glycosyl-phosphatidylinositol (GPI) anchor, and is an IFN-induced type II transmembrane protein (Zhao et al., 2022b). BST2 inhibits the release process of various viruses (Andrew et al., 2009). Studies have shown that BST2 can activate the proinflammatory factor NF-κB by recruiting TRAF2 and/or TRAF6, TAB, and the mitogen-activated kinase MAP3K7/TAK1 (Tokarev et al., 2013). BST2 inhibits influenza A virus infection by promoting the apoptosis of infected cells (Yi et al., 2019). BST2 can inhibit type I IFN signaling by recruiting the E3 ubiquitin ligase MARCHF8 to catalyze the conversion of ubiquitin chains to mitochondrial antiviral-signaling proteins (MAVs) for degradation. Results from a previous study in our laboratory found that BST2 inhibits the replication of porcine epidemic diarrhea virus (PEDV) by delivering the PEDV N protein to autophagosomes for selective degradation (Kong et al., 2020). In this study, we aimed to evaluate whether interferon-induced BST2 can inhibit the replication of PRRSV and analyze the effects of BST2 overexpression and knockdown on PRRSV replication. We also aimed to identify the antiviral mechanism of BST2, which added another layer of complexity to the innate antiviral immunity of host restriction factor against PRRSV.

## 2 Materials and methods

### 2.1 Cells and virus

Porcine alveolar macrophages (PAMs), MARC-145, and 293T cells (ATCC, USA) were cultured as previously described (Zhang et al., 2020). The highly pathogenic PRRSV HuN4 (GenBank accession No. EF635006) were stored in our laboratory (Tong et al., 2007).

### 2.2 Plasmid construction

According to the nucleotide information of porcine *BST2* gene (GenBank accession no. KC346970.1), BST2-encoding sequences were amplified and cloned into p3×Flag or pCAGGS vector to generate p3×Flag-BST2 and pCAGGS-BST2-HA as previously described (Kong et al., 2020). The mutant vectors (p3×Flag-BST2-N68A, p3×Flag-BST2-N95A, and p3×Flag-BST2-N68N95A) were generated from wild-type p3×Flag-BST2 via site-directed mutagenesis. Sequences of all primers used for gene amplification are available upon request.

### 2.3 Over-expression and knocking down of BST2 on PRRSV replication

MARC-145 cells were cultured in six-well plates. When the cells reached approximately 60% confluence, they were transfected with p3×Flag/or p3×Flag-BST2, siRNA NC, or siRNA BST2. After 36 h, cells were infected with HuN4 at an multiplicity of infection (MOI) of 0.05 or 0.1. The cells were washed thrice with PBS. Each sample was collected using 200 μL RIPA lysis buffer supplemented with protease and phosphatase inhibitors (Bimake, Shanghai, China,1:100) on ice. To each sample, a 5×SDS loading buffer was added and boiled for 10 min. Western blotting and RT-qPCR were used to analyze the effect of BST2 overexpression and knockdown on PRRSV replication.

### 2.4 Western blotting

Cell lysates were fractionated using sodium dodecyl sulfate-polyacrylamide gel and blotted onto nitrocellulose membranes. The membranes were blocked for 1 h using 5% nonfat milk and probed with specific primary antibodies for 2 h. After the membranes were washed with TBST thrice, they were incubated in the corresponding HRP IgG(*H*+*L*) secondary antibody (SA00001, Proteintech Group, Rosemont, IL, USA) for 1 h. Signals were detected using an enhanced chemiluminescence kit, Tanon-5200 automatic chemiluminescence image analysis system. The primary antibodies used were as follows: mouse anti-β actin monoclonal antibody (Sigma-Aldrich, St. Louis, MO, USA, 1:6000), mouse anti-Flag monoclonal antibody (Sigma-Aldrich, 1:1000), mouse anti-HA monoclonal antibody (Sigma-Aldrich, 1:6000), mouse anti-Myc monoclonal antibody (Cell Signaling Technology, Danvers, MA, USA, 1:1000), mouse anti-N polyclonal antibody (prepared in our lab, 1:1000), and mouse anti-BST2 monoclonal antibody (prepared in our lab, 1:1000).

### 2.5 RNA extraction and RT-qPCR assay

The total RNA of cells was extracted using a RNeasy Mini Kit (QIAGEN, Hilden, Germany, 74,104) and the RNA of the supernatant was extracted using a QIAamp Viral RNA Mini Kit (QIAGEN, 52,906). RNA was then reverse-transcribed into cDNA using a reverse transcriptase mix (Takara, Dalian, China). STBR Premix Ex Taq™ was used for RT-qPCR. Monkey BST2 (Kong et al., 2020) and GAPDH primer sequences (Zhang et al., 2020) were designed as previously described.

### 2.6 TCID_50_ assay for PRRSV

MARC-145 cells were seeded in 96-well plates and infected with 10-fold serial dilutions of PRRSV samples for 5 d (eight replicates per dilution). Virus titers were calculated based on the Reed–Muench method (Zhang et al., 2020).

### 2.7 Co-immunoprecipitation

293T cells were co-transfected with the indicated plasmids in duplicate using Lipofectamine 3000 transfection kit (Thermo Fisher Scientific, Waltham, MA, USA). Twenty-four hours after transfection, each sample was collected using 200 μL IP lysis buffer supplemented with protease inhibitor and phosphatase inhibitor (Bimake). A 50 μL aliquot of lysate was taken for input detection, and 350 μL of lysate was added to 15 μL of agarose beads with monoclonal antibody against Myc and 250 μL IP lysis buffer at 4 °C for incorporation. Six hours later, the beads were pelleted and washed five times with IP lysis buffer. Finally, the beads were resuspended in 50 μL IP lysis buffer and boiled for 10 min with 5×SDS loading buffer.

### 2.8 Virus adsorption experiments

MARC-145 cells were infected with HuN4 at an MOI of 0.1 at 4 °C for 1 h, the supernatant was discarded, and the cells were washed with cold PBS thrice. Total RNA was collected using an RNeasy Mini Kit (QIAGEN) and reverse-transcribed into cDNA with a reverse transcriptase mix (Takara). STBR Premix Ex Taq™ was used for RT-qPCR.

### 2.9 Virus entry experiments

MARC-145 cells were infected with HuN4 at an MOI of 0.1 at 37 °C for 1 h. The supernatant was discarded, and the cells were washed with cold PBS thrice. Total RNA was collected using an RNeasy Mini Kit (QIAGEN) and reverse-transcribed into cDNA using a reverse transcriptase mix (Takara). SYBR Premix Ex Taq™ was used for RT-qPCR analysis.

### 2.10 Statistical analysis

All experiments were performed at least thrice independently. Data were analyzed using GraphPad Prism 5, and statistical significance was analyzed using the unpaired two-tailed Student's *t*-test. *, P<0.05, **, p<0.01, ***, P<0.001. The gray values from western blotting were calculated using ImageJ software.

## 3 Results

### 3.1 Over-expression of BST2 inhibited the replication of prrsv in MARC-145 cells

**Fig. 1:**
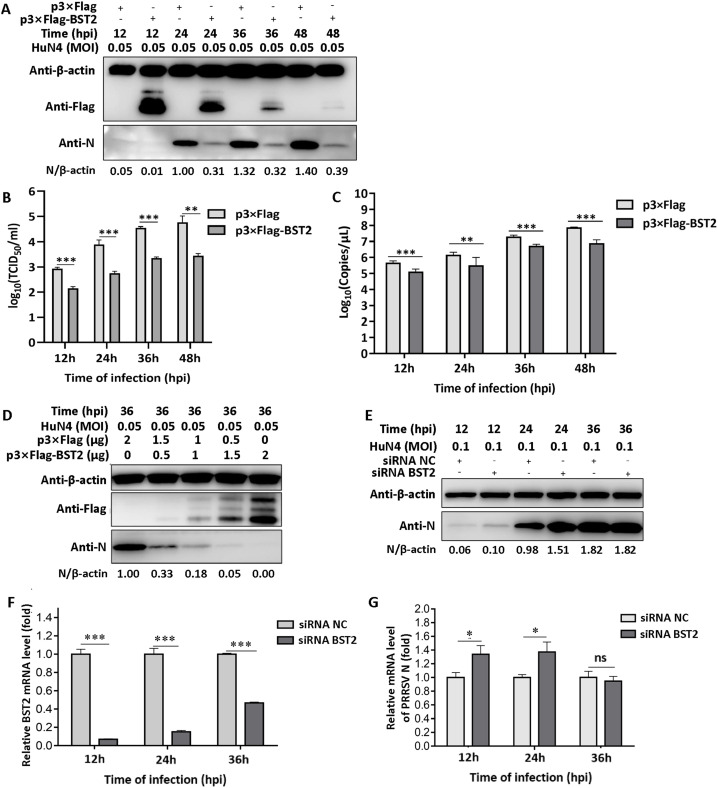
Inhibition of the expression of PRRSV by BST2 (A) PRRSV N protein levels in MARC-145 cells transfected with p3×Flag-BST2 (2.5 μg) or pCAGGS (2.5 μg) at 12, 24, 36, and 48 hpi via western blot analysis. (B) Viral titers from MARC-145 cells transfected with p3×Flag-BST2 (2.5 μg) or pCAGGS (2.5 μg). Supernatants were collected at the indicated times and titrated. (C) Viral copies of PRRSV N protein from MARC-145 cells transfected with p3×Flag-BST2 (2.5 μg) or pCAGGS (2.5 μg). Supernatants were collected at the indicated times and analyzed via RT-qPCR. (D) MARC-145 cells were transfected with different doses of BST2 for 36 h, infected with HuN4 at a MOI of 0.05, and collected at 36 hpi. N protein was analyzed via western blotting. (E) MARC-145 cells were transfected with siRNA NC or siRNA BST2 (100 mM) for 36 h and infected with PRRSV at an MOI of 0.1. PRRSV N protein levels were analyzed via western blotting at 12 hpi, 24 hpi, and 36 hpi. The values below were calculated from N protein gray values against the corresponding β-actin gray values. (F) MARC-145 cells were transfected with siRNA BST2 (100 mM), and the knockdown efficiency of the siRNAs was analyzed at 12, 24, and 36 h. (G) Relative mRNA levels of PRRSV N protein in MARC-145 cells transfected with siRNA NC or siRNA BST2 at 12, 24, and 36 hpi. Data represent the mean±standard deviation (SD) of three independent experiments. Statistical significance was analyzed using *t*-test. *P < 0.05; **P < 0.01; ***P < 0.001.

BST2, an IFN-inducible gene, can be induced via multiple stimuli and plays an important role in the antiviral response. BST2 can inhibit the replication of multiple viruses (Zhao et al., 2022b). To investigate whether BST2 inhibits PRRSV replication, the DNA sequences of porcine *BST2* and monkey *BST2* were cloned into a p3×Flag vector (Kong et al., 2020). PAMs are the target cells of PRRSV *in vivo* and MARC-145 cells are PRRSV-susceptible cells *in vitro*. Owing to the low transfection efficiency in primary cells, BST2 was transfected into MARC-145 cells for 36 h, and then infected with PRRSV at an MOI of 0.05. Whole cell lysates were collected for western blotting (Fig. 1A); the N protein levels of PRRSV were significantly lower in MARC-145 cells previously transfected with p3×Flag-BST2 than those in cells transfected with the empty vector. The supernatants were collected at different times to detect viral titers and loads and to investigate the role of BST2 in the replication of PRRSV. As expected, viral titers and loads were significantly lower in MARC-145 cells previously transfected with p3×Flag-BST2 than those in cells transfected with the empty vector (Fig. 1B and 1C). The inhibitory capability of BST2 correlated to expression levels of BST2, and increasing the dose of BST2 further decreased the viral ORF7 levels (Fig. 1D).

To further investigate the role of BST2 in PRRSV replication, siRNAs were designed to knock down the endogenous expression of *BST2*, and the cells were then infected with PRRSV at an MOI of 0.1. Western blotting and RT-qPCR were used to analyze the extent of PRRSV replication. We observed that knocking down the expression of BST2 increased the replication of PRRSV at 12 and 24 h post infection (hpi); however, the replication of PRRSV was not significantly different between cells transfected with siRNA NC and siRNA BST2 at 36 hpi, as indicated by western blotting results (Fig. 1E). To investigate the lack of difference between siRNA NC and siRNA BST2 at 36 hpi, siRNA NC and siRNA BST2 were transfected into MARC-145 cells infected with PRRSV at an MOI of 0.1, and the total RNA was collected. BST2 and PRRSV N protein mRNA levels were analyzed using RT-qPCR (Fig. 1F and 1G). As shown in Fig. 1F, the mRNA expression of BST2 was still lower in cells transfected with siRNA BST2 than that in cells transfected with siRNA NC at 36 hpi; however, the efficiency of knocking down BST2 was lower at 36 hpi than that at 12 and 24 hpi. Based on these results, we can conclude that BST2 inhibited the replication of PRRSV and that PRRSV might counteract the antiviral activities of BST2 via a specific mechanism.

### 3.2 Upregulated expression of BST2 in the early stage of PRRSV infection in PAMs and MARC-145 cells

**Fig. 2:**
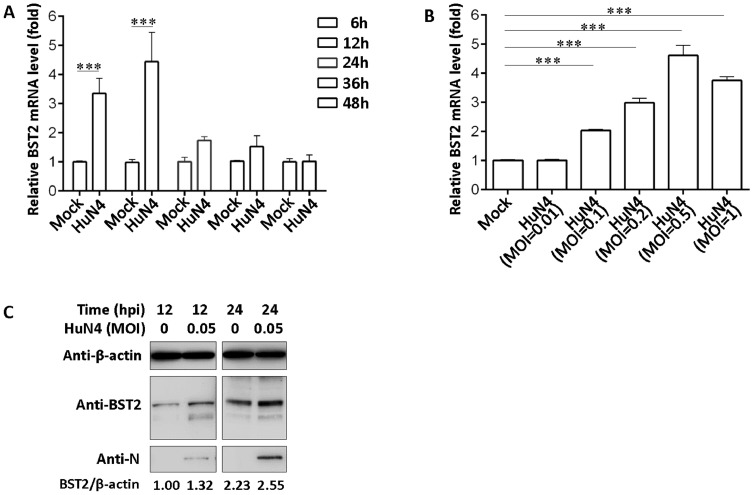
BST2 mRNA and PAM protein expression with or without PRRSV infection (A) PAMs were infected with PRRSV HuN4 at a MOI of 0.5, and the cells were harvested at 6, 12, 24, 36, and 48 h, and BST2 mRNA levels were monitored via qRT-PCR. (B) PAMs were infected with PRRSV HuN4 of different MOI, and the cells were harvested at 12 hpi, and BST2 mRNA levels were monitored via qRT-PCR. Data represent the mean±SD of three independent experiments. Statistical significance was analyzed using t-test. ***P < 0.001. (C) PAMs were infected with PRRSV at a MOI of 0.05, and the sample was collected at 12 and 24 hpi for detecting the expression of endogenous BST2.

To investigate the expression of BST2 following PRRSV infection, we determined the expression of endogenous BST2 in PAMs using RT-qPCR and western blotting. PAMs were infected with PRRSV at an MOI of 0.5, and total RNAs of PAMs were collected for endogenous BST2 mRNA detection. Endogenous mRNA of BST2 was significantly upregulated at 6 and 12 hpi and slightly upregulated at 24, 36, and 48 hpi (Fig. 2A). Moreover, BST2 mRNA expression increased in a dose-dependent manner 12 h after PRRSV infection (Fig. 2B). The expression of endogenous BST2 protein was also upregulated at low doses of PRRSV infection in PAMs (Fig. 2C), indicating that PRRSV infection upregulated the expression of BST2 *in vitro*.

### 3.3 BST2 inhibited the post-entry stage of PRRSV replication

**Fig. 3:**
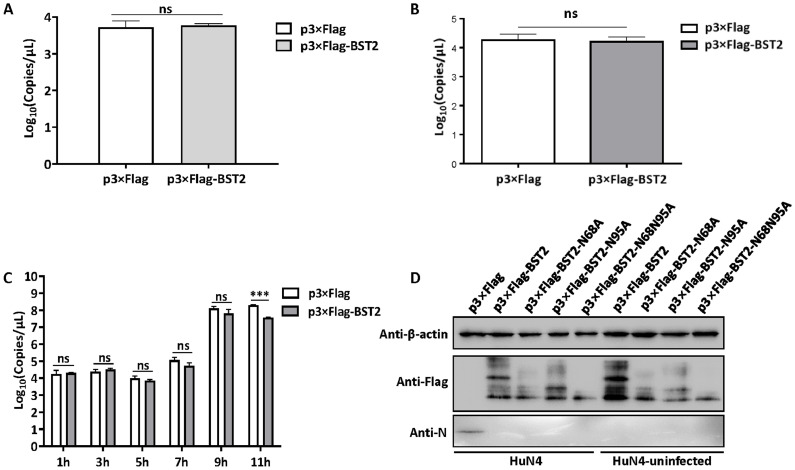
BST2 inhibits the post-entry process of PRRSV (A) Cells were incubated at 4 °C for 1 h and total RNA was collected. RT-qPCR was used for detecting the PRRSV N protein. (B) The cells were incubated at 37 °C for the indicated times, total RNA was collected, and RT-qPCR was performed for detecting the mRNA levels of N protein. Data represent the mean±standard deviation (SD) of three independent experiments. Statistical significance was analyzed using t-test. ***P < 0.001; ns, not significant. (C) MARC-145 cells were transfected with p3×Flag-BST2 (2.5 μg) or p3×Flag (2.5 μg). and incubated with PRRSV for different durations. Total RNA was collected at the indicated time, and RT-qPCR was performed for detecting the mRNA levels of N protein. (D) MARC-145 cells were transfected with 2.5 μg of either p3×Flag, p3×Flag-BST2 or its mutations for 36 h, and then were infected with HuN4 at a MOI of 0.1. Cell lysates were harvested at 24 hpi, and western blotting was performed using an antibody against N protein.

BST2 inhibits viral replication by tethering mature envelope viruses to the cell surface and inhibiting the virus release process. Meanwhile, BST2 enhances the entry of human cytomegalovirus (Viswanathan et al., 2011). To investigate the role of BST2 in PRRSV replication, a series of experiments were performed. BST2 did not exert any effect on the attachment or entry processes of PRRSV replication (Fig. 3A and 3B). We collected the total RNA of MARC-145 cells that were transfected with BST2 and incubated with PRRSV for different durations and found that BST2 had no impact on PRRSV replication until 11 hpi (Fig. 3C), indicating that BST2 was involved in the post-entry process of PRRSV.

The glycosylation of BST2 plays a vital role in antiviral activity. SARS-CoV ORF7a inhibits BST2 glycosylation, and unglycosylated BST2 no longer restricts viral release, leading to a loss in the antiviral function of BST-2 (Taylor et al., 2015). Human BST2 contains two residues (N65 and N92) that are required for its glycosylation (Andrew et al., 2009), and mutants of the BST2 glycosylation site lose their ability to inhibit viral release. As shown in Fig. 3D, we found that the mutants of the BST2 glycosylation site still had the ability to inhibit viral release, indicating that the glycosylation of BST2 was not essential for its inhibitory effect on PPRSV.

### 3.4 BST2 inhibited the transcription of PRRSV E protein

**Fig. 4:**
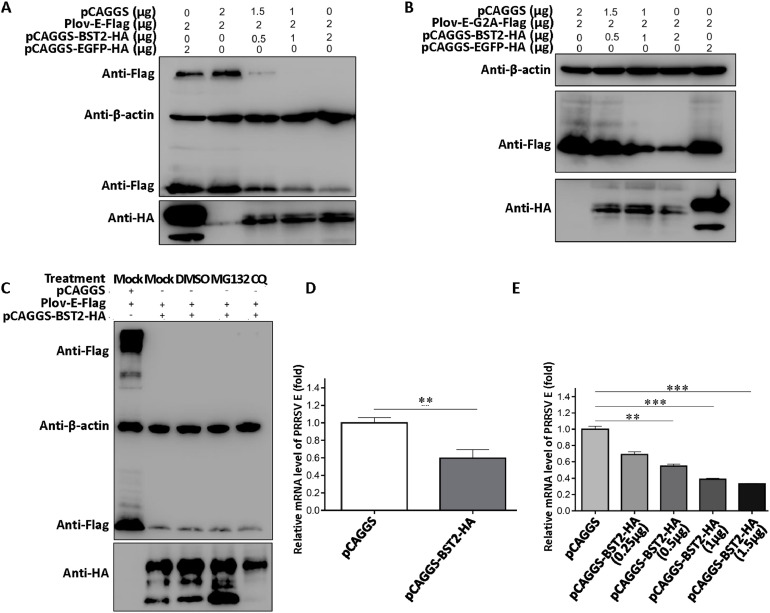
BST2 can inhibit the transcription of PRRSV E protein (A) 293T cells were transfected with pCAGGS-BST2-HA or pCAGGS-EGFP-HA and pLov-E-Flag for 24 h, and proteins were collected and analyzed via western blotting. (B) 293T cells were transfected with pCAGGS-BST2-HA or pCAGGS-EGFP-HA and pLov-E-G2A-Flag for 24 h, and proteins were collected and analyzed via western blotting. (C) 293T cells were transfected with pLov-E-Flag and pCAGGS-BST2-HA for 18 h, and treated with DMSO, MG132, or CQ for 6 h. Proteins were then collected and analyzed via western blotting. (D) 293T cells were transfected with pCAGGS or pCAGGS-BST2-HA and pLov-E-Flag for 24 h. Total RNA was collected and analyzed via RT-qPCR. (E) 293T cells were co-transfected with different doses of BST2 and pLov-E-Flag for 24 h, and whole RNA was collected and analyzed via western blotting. Data represent the mean±SD of three independent experiments. Statistical significance was analyzed using t-test. **P < 0.01; ***P < 0.001.

BST2 inhibits the replication of PRRSV at an early stage, although PRRSV might have the ability to counteract the antiviral activities of BST2. When BST2 interacts with the E protein of PRRSV, E protein downregulates the expression of BST2, which may be one mechanism of counteracting the antiviral activity of BST2 (Wang et al., 2014). We further investigated the effect of BST2 on the E protein. Results showed that BST2 downregulated the expression of E protein in a dose-dependent manner (Fig. 4A). The E protein contains the consensus motif for myristoylation, and N-terminal myristoylation refers to the linkage of myristic acid (C14:O) via an amide bond to the N-terminal glycine residue of a cellular, viral, or a bacterial protein (Boutin, 1997; James and Olson, 1990; Nimchuk et al., 2000; Wilcox et al., 1987). The G2A mutant of PRRSV E protein can decrease virus titers; however, it is not essential for PRRSV infectivity (Du et al., 2010). Based on this, we speculated whether the decreased expression of the E protein was related to the myristoylation of BST2. A G2A mutant of E protein was constructed. Our results showed that BST2 treatment downregulated the expression of PRRSV E protein (Fig. 4B). Hence, we hypothesized that the decrease in E protein by BST2 was derived from the transcriptional and translational levels.

The ubiquitin–proteasome and lysosomal pathways are the two main pathways involved in protein degradation. As shown in Fig. 4C, the ubiquitin proteasome inhibitor MG132 and lysosomal inhibitor chloroquine (CQ) did not restore the expression of E protein, establishing that the decrease in E protein by BST2 was not due to post-translational modification levels. Further expression tests showed that BST2 inhibited the transcription of E protein and decreased E protein production in a dose-dependent manner (Fig. 4D and 4E).

### 3.5 BST2 inhibited the expression of most non-structural proteins of PRRSV

**Fig. 5:**
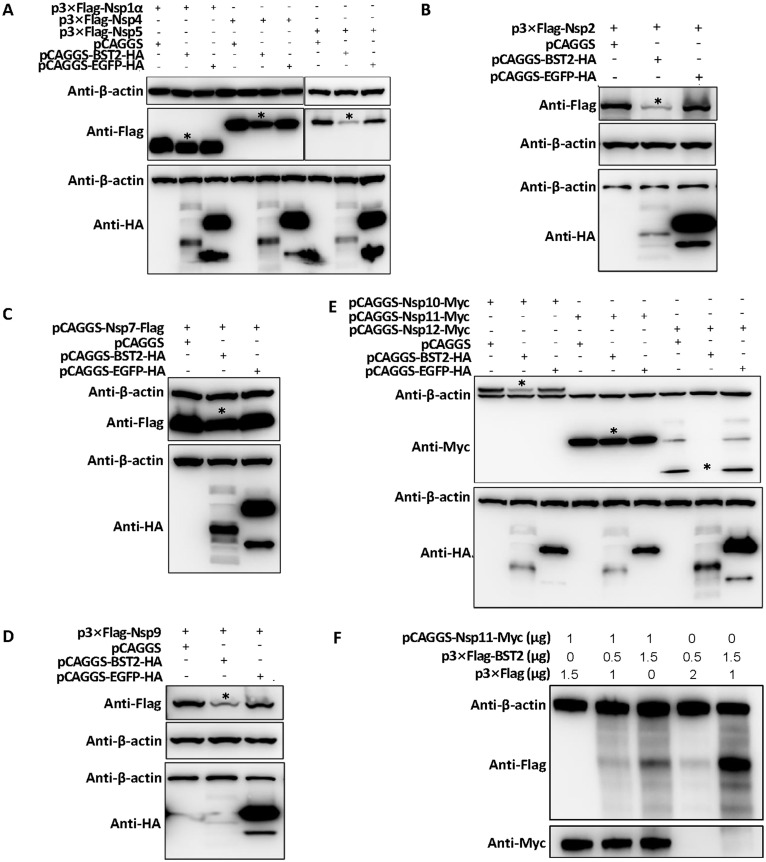
BST2 can inhibit the expression of the non-structural proteins of PRRSV, except Nsp11 Different non-structural proteins were co-transfected with the empty vector pCAGGS, pCAGGS-BST2-HA, or pCAGGS-EGFP-HA in 293T cells. After 24 hpi, whole proteins were collected and analyzed via western blotting. The non-structural proteins were as follows: (A) Nsp1α, Nsp4, and Nsp5 (B) Nsp2 (C) Nsp7 (D) Nsp9 (E) Nsp10, Nsp11, and Nsp12 (F) BST2 could not inhibit the expression of PRRSV Nsp11; however Nsp11 inhibited the expression of BST2.

To further explore the role of BST2 on PRRSV replication, the following non-structural proteins of PRRSV were constructed; Nsp1, Nsp2, Nsp4, Nsp5, Nsp7, Nsp9, Nsp10, Nsp11, and Nsp12. The same doses of different non-structural proteins of PRRSV were co-transfected with the empty vectors, pCAGGS, pCAGGS-HA-BST2, and pCAGGS-HA-EGFP separately. EGFP was used as a parallel control to confirm the specificity of BST2. Results showed that BST2 can inhibit the expression of Nsp1, Nsp4, Nsp5 (Fig. 5A), Nsp2 (Fig. 5B), Nsp7 (Fig. 5C), Nsp9 (Fig. 5D), Nsp10, and Nsp12, but has little impact on the expression of Nsp11 (Fig. 5E). An increase in BST2 did not decrease the expression of Nsp11 (Fig. 5F). BST2 inhibited the expression of most non-structural proteins of PRRSV, except PRRSV Nsp11.

### 3.6 BST2 inhibited the expression of nsp12 by ubiquitin–proteasome pathway

**Fig. 6:**
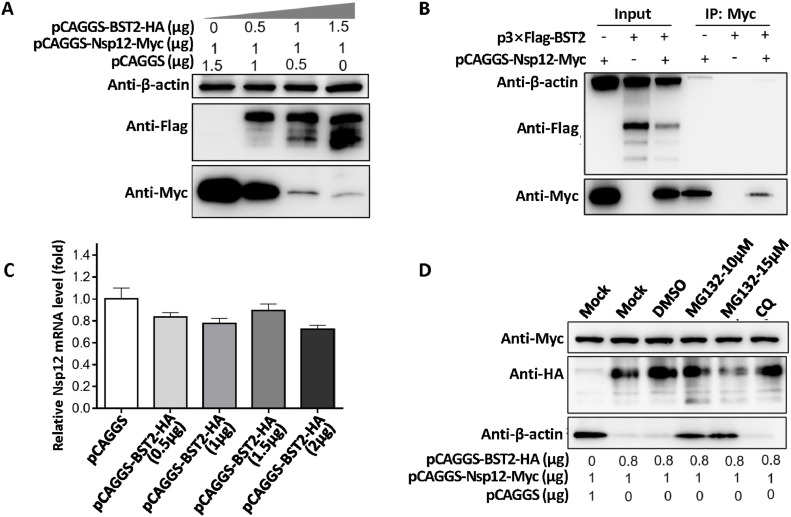
BST2 inhibits PRRSV Nsp12 through a proteasome-dependent pathway. (A) 293T cells were co-transfected with pCAGGS-Nsp12-Myc (1 µg) and increasing amounts of pCAGGS-BST2-HA (0, 0.5, 1, or 1.5 µg), and Nsp12 levels were determined using western blotting. (B) 293T cells were co-transfected with pCAGGS-BST2-HA and pCAGGS-Nsp12-Myc. Cell lysates were precipitated with an anti-Myc monoclonal antibody and separated using a protein A-sepharose column for assessment via western blotting, with β-actin used as a loading control. (C) 293T cells were co-transfected with pCAGGS-Nsp12-Myc and increasing doses of pCAGGS-BST2-HA (0, 0.5, 1, or 1.5 µg). The expression level of Nsp12 was detected via RT-qPCR. (D) BST2 promotes autophagic degradation of Nsp12. pCAGGS-Nsp12-Myc was co-transfected with pCAGGS or pCAGGS-BST2-HA into 293T cells, which were then treated for 12 h with MG132 or CQ. Nsp12 levels were determined via western blotting using anti-Myc antibody.

As BST2 can inhibit most non-structural proteins of PRRSV, and the role of Nsp12 is particularly significant, we further investigated the degradative mechanism of BST2 on PRRSV Nsp12. Results showed that the degradation of Nsp12 by BST2 occurred in a dose-dependent manner (Fig. 6A), and BST2 could not interact with PRRSV Nsp12 (Fig. 6B). Meanwhile, BST2 had no effect on the transcription level of Nsp12 (Fig. 6C). The ubiquitin proteasome inhibitor (MG132) restored expression, whereas CQ could not restore the expression of Nsp12 (Fig. 6D).

## 4 Discussion

In this study, we found that BST2 inhibited the replication of PRRSV. During early stages of PRRSV infection, the expression of BST2 was upregulated to inhibit the replication of PRRSV. However, the expression of BST2 was downregulated when infected with a large dose of PRRSV. We further identified the antiviral mechanism of BST2, which demonstrated the complex crosstalk between BST2 and PRRSV replication.

The immune response to PRRSV infection is poor, and the antiviral and antagonistic mechanisms of host proteins are important for the control of PRRSV, especially in cases where the protective abilities of commercial vaccines are limited to circulating viruses (Chang et al., 2019; Dong et al., 2018; Jin et al., 2017a; Ke et al., 2019; Song et al., 2019). In this study, we showed that porcine BST2 overexpression inhibited PRRSV replication in a dose-dependent manner, whereas *BST2* knockdown facilitated PRRSV replication (Fig. 1), suggesting that BST2 could be a potential target for controlling PRRSV infection.

A recent study in our laboratory found that BST2 can suppress porcine epidemic diarrhea virus replication by targeting and degrading the viral nucleocapsid protein with selective autophagy (Kong et al., 2020), which broadens the antiviral mechanism of BST2. However, the underlying mechanism by which BST2 affects PRRSV replication is not well understood. In the present study, we found that PRRSV upregulated the transcription and translation of BST2 at an early stage of PRRSV infection in PAMs (Fig. 2). Upregulation of endogenous BST2 expression decreased with prolonged duration of PRRSV infection *in vitro* (Fig. 2), suggesting important antiviral roles of BST2 during the early stage of PRRSV infection. Host cells appear to actively upregulate BST2 expression in response to PRRSV infection, and PRRSV might have the ability to antagonize the antiviral effect of BST2, which highlights the complex interplay between host and PRRSV.

BST2 is an interferon-inducible transmembrane protein that participates in antiviral defense in various ways, such as by inhibiting HIV release (Van Damme et al., 2008). Apart from its role as a viral tether, BST2 can activate NF-κB pathaway to fend off host pathogen infections (Kong et al., 2020; Tokarev et al., 2013). Jin et al. suggested that BST2 can degrade MAV_S_ via autophagy (Jin et al., 2017b) and degrade the nucleocapsid protein of porcine epidemic diarrhea virus via selective autophagy (Kong et al., 2020). In addition, recent studies have found that BST2 can suppress LINE-1 retro transposition by reducing the promoter activity of LINE-1 5′ UTR (Zhao et al., 2022a). The relationship between BST2 and viruses is multifaceted, and viruses have developed several ways to antagonize the antiviral effects of BST2. For instance, HIV-1 Vpu can counteract the signal activation of NF-κB (Tokarev et al., 2013). HIV-1 can block recycling and biosynthetic transport of the intrinsic BST2 (Schmidt et al., 2011) and promote sorting of BST2 for lysosomal degradation through recruitment of the E3 ligase complex SCF adaptors, β-TrCP1 and β-TrCP2 (Roy et al., 2017). Moreover, BST2 is consisted of a short N-terminal cytoplasmic domain, an α-helical TM domain, an extracellular domain containing an a-helical CC domain, and a C-terminal GPI anchor. The conserved YxY motif of NT domain is important for the activation of NF-κB. Three cysteine residues and two glycosylated residues are important for the antiviral activity of BST2 (Zhao et al., 2022b). In this study, we found that BST2 did not exert any effect on the adsorption and entry stages of PRRSV replication and played a role in the post-entry stage (Fig. 3A–C). To determine the effect of BST2 glycosylation on PRRSV replication, we constructed mutants of single and total glycosylation and found that all mutants still exhibited antiviral activity (Fig. 3D). In addition, some researchers have found that cysteine-linked dimers of BST2 are critical for the inhibition of HIV-1 virus release. Only BST2 lacking all three cysteine residues lost its antiviral activity, whereas BST2 that retained any one of the three cysteine residue still exhibited an antiviral effect (Andrew et al., 2009).

We assessed the possible interactions between BST2 and PRRSV Nsp and E proteins. As a previous study showed that BST2 could interact with the E protein of PRRSV (Wang et al., 2014), we further investigated the outcome of this interaction and found that BST2 could inhibit the expression of E protein at the transcription level instead of the translation level. The decrease in E protein mRNA was significantly dependent on the dose of BST2 (Fig. 4); however, the underlying mechanism requires further investigation. BST2 was found to clearly inhibit the expression of most PRRSV Nsps, except Nsp11, whereas the EGF protein did not inhibit the expression of PRRSV Nsps (Fig. 5). Considering the significant effect of BST2 on Nsp12, further evaluation found that BST2 can downregulate the expression of Nsp12 via the ubiquitin–proteasome pathway (Fig. 6). To explain the ability of BST2 to inhibit the expression of most Nsps other than Nsp11, we hypothesized that it may be related to an antagonistic mechanism of PRRSV and that the Nsp11 of PRRSV may use its endonuclease to downregulate the expression of BST2 and reduce antiviral activity (data not shown here).

In summary, we demonstrated that BST2 exhibited significant anti-PRRSV activity by restricting the expression of viral proteins via different mechanisms. These findings demonstrate the potential of BST2 as a critical regulator of PRRSV replication. The findings of this study would effectively guide further investigations into the role of BST2 in resistance to PRRS transmission, thus broadening our understanding of host–PRRSV regulatory mechanisms.

## Author statement

All authors have contributed to, seen and approved the final and submitted version. We have no conflicts of interest to disclose.

## Declaration of Competing Interest

There are no conflicts of interest to declare.

## Data Availability

Data will be made available on request.

## References

[bib0001] An T.Q., Li J.N., Su C.M., Yoo D. (2020). Molecular and cellular mechanisms for PRRSV pathogenesis and host response to infection. Virus Res..

[bib0002] Andrew A.J., Miyagi E., Kao S., Strebel K. (2009). The formation of cysteine-linked dimers of BST-2/tetherin is important for inhibition of HIV-1 virus release but not for sensitivity to Vpu. Retrovirology.

[bib0004] Boutin J.A. (1997). Myristoylation. Cell Signal.

[bib0005] Chang X., Shi X., Zhang X., Wang L., Li X., Wang A., Deng R., Zhou E., Zhang G. (2019). IFI16 inhibits porcine reproductive and respiratory syndrome virus 2 replication in a MAVS-dependent manner in MARC-145 cells. Viruses.

[bib0006] Dokland T. (2010). The structural biology of PRRSV. Virus Res..

[bib0007] Dong H., Zhou L., Ge X., Guo X., Han J., Yang H. (2018). Porcine reproductive and respiratory syndrome virus nsp1beta and nsp11 antagonize the antiviral activity of cholesterol-25-hydroxylase via lysosomal degradation. Vet. Microbiol..

[bib0008] Du T., Nan Y., Xiao S., Zhao Q., Zhou E.M. (2017). Antiviral strategies against PRRSV infection. Trends Microbiol..

[bib0009] Du Y., Zuckermann F.A., Yoo D. (2010). Myristoylation of the small envelope protein of porcine reproductive and respiratory syndrome virus is non-essential for virus infectivity but promotes its growth. Virus Res..

[bib0010] Fang Y., Snijder E.J. (2010). The PRRSV replicase: exploring the multifunctionality of an intriguing set of nonstructural proteins. Virus Res..

[bib0011] James G., Olson E.N. (1990). Fatty acylated proteins as components of intracellular signaling pathways. Biochemistry.

[bib0012] Jin H., Zhou L., Ge X., Zhang H., Zhang R., Wang C., Wang L., Zhang Z., Yang H., Guo X. (2017). Cellular DEAD-box RNA helicase 18 (DDX18) promotes the PRRSV replication via interaction with virus nsp2 and nsp10. Virus Res..

[bib0013] Jin S., Tian S., Luo M., Xie W., Liu T., Duan T., Wu Y., Cui J. (2017). Tetherin suppresses type I interferon signaling by targeting MAVS for NDP52-mediated selective autophagic degradation in human cells. Mol. Cell.

[bib0014] Ke W., Fang L., Tao R., Li Y., Jing H., Wang D., Xiao S. (2019). Porcine reproductive and respiratory syndrome virus E protein degrades porcine cholesterol 25-hydroxylase via the ubiquitin-proteasome pathway. J. Virol..

[bib0015] Kong N., Shan T., Wang H., Jiao Y., Zuo Y., Li L., Tong W., Yu L., Jiang Y., Zhou Y., Li G., Gao F., Yu H., Zheng H., Tong G. (2020). BST2 suppresses porcine epidemic diarrhea virus replication by targeting and degrading virus nucleocapsid protein with selective autophagy. Autophagy.

[bib0016] Li L., Bai Y., Zhou Y., Jiang Y., Tong W., Li G., Zheng H., Gao F., Tong G. (2023). PSMB1 inhibits the replication of porcine reproductive and respiratory syndrome virus by recruiting NBR1 to degrade nonstructural protein 12 by autophagy. J. Virol..

[bib0017] Li L., Zhao K., Gao F., Jiang Y., Shan T., Tong W., Zheng H., Yu L., Li G., Ma Z., Tong G. (2019). Restriction of porcine reproductive and respiratory syndrome virus replication by galectin-1. Vet. Microbiol..

[bib0018] Lunney J.K., Fang Y., Ladinig A., Chen N., Li Y., Rowland B., Renukaradhya G.J. (2016). Porcine reproductive and respiratory syndrome virus (PRRSV): pathogenesis and Interaction with the immune system. Annu Rev Anim Biosci.

[bib0019] Nimchuk Z., Marois E., Kjemtrup S., Leister R.T., Katagiri F., Dangl J.L. (2000). Eukaryotic fatty acylation drives plasma membrane targeting and enhances function of several type III effector proteins from Pseudomonas syringae. Cell.

[bib0020] Roy N., Pacini G., Berlioz-Torrent C., Janvier K. (2017). Characterization of E3 ligases involved in lysosomal sorting of the HIV-1 restriction factor BST2. J. Cell Sci..

[bib0021] Schmidt S., Fritz J.V., Bitzegeio J., Fackler O.T., Keppler O.T. (2011). HIV-1 Vpu blocks recycling and biosynthetic transport of the intrinsic immunity factor CD317/tetherin to overcome the virion release restriction. MBio.

[bib0022] Song Z., Bai J., Liu X., Nauwynck H., Wu J., Liu X., Jiang P. (2019). S100A9 regulates porcine reproductive and respiratory syndrome virus replication by interacting with the viral nucleocapsid protein. Vet. Microbiol..

[bib0023] Taylor J.K., Coleman C.M., Postel S., Sisk J.M., Bernbaum J.G., Venkataraman T., Sundberg E.J., Frieman M.B. (2015). Severe acute respiratory syndrome coronavirus ORF7a inhibits bone marrow stromal antigen 2 virion tethering through a novel mechanism of glycosylation interference. J. Virol..

[bib0024] Tokarev A., Suarez M., Kwan W., Fitzpatrick K., Singh R., Guatelli J. (2013). Stimulation of NF-kappaB activity by the HIV restriction factor BST2. J. Virol..

[bib0025] Tong G.Z., Zhou Y.J., Hao X.F., Tian Z.J., An T.Q., Qiu H.J. (2007). Highly pathogenic porcine reproductive and respiratory syndrome, China. Emerg. Infect. Dis..

[bib0026] Van Damme N., Goff D., Katsura C., Jorgenson R.L., Mitchell R., Johnson M.C., Stephens E.B., Guatelli J. (2008). The interferon-induced protein BST-2 restricts HIV-1 release and is downregulated from the cell surface by the viral Vpu protein. Cell Host Microbe.

[bib0027] Viswanathan K., Smith M.S., Malouli D., Mansouri M., Nelson J.A., Fruh K. (2011). BST2/Tetherin enhances entry of human cytomegalovirus. PLoS Pathog..

[bib0028] Wang X., Li C., Zhou L., Zhang N., Wang X., Ge X., Guo X., Yang H. (2014). Porcine reproductive and respiratory syndrome virus counteracts the porcine intrinsic virus restriction factors-IFITM1 and Tetherin in MARC-145 cells. Virus Res..

[bib0029] Wilcox C., Hu J.S., Olson E.N. (1987). Acylation of proteins with myristic acid occurs cotranslationally. Science.

[bib0030] Yi E., Oh J., Kang H.R., Song M.J., Park S.H. (2019). BST2 inhibits infection of influenza A virus by promoting apoptosis of infected cells. Biochem. Biophys. Res. Commun..

[bib0031] Zhang Y., Gao F., Li L., Zhao K., Jiang S., Jiang Y., Yu L., Zhou Y., Liu C., Tong G. (2020). Porcine reproductive and respiratory syndrome virus antagonizes PCSK9′s antiviral effect via Nsp11 endoribonuclease activity. Viruses.

[bib0032] Zhao K., Li L.W., Zhang Y.J., Jiang Y.F., Gao F., Li G.X., Yu L.X., Zhao W.Y., Shan T.L., Zhou Y.J., Tong G.Z. (2018). MOV10 inhibits replication of porcine reproductive and respiratory syndrome virus by retaining viral nucleocapsid protein in the cytoplasm of Marc-145 cells. Biochem. Biophys. Res. Commun..

[bib0033] Zhao Y., Du J., Wang Y., Wang Q., Wang S., Zhao K. (2022). BST2 suppresses LINE-1 retrotransposition by reducing the promoter activity of line-1 5′ UTR. J. Virol..

[bib0034] Zhao Y., Song Z., Bai J., Liu X., Nauwynck H., Jiang P. (2020). Porcine reproductive and respiratory syndrome virus Nsp4 cleaves ZAP to antagonize its antiviral activity. Vet. Microbiol..

[bib0035] Zhao Y., Zhao K., Wang S., Du J. (2022). Multi-functional BST2/tetherin against HIV-1, other viruses and LINE-1. Front. Cell Infect. Microbiol..

